# The trends for the “trend toward significance” in the pediatric literature

**DOI:** 10.1007/s00431-022-04746-8

**Published:** 2022-12-02

**Authors:** Dimitrios Rallis, Maria Baltogianni, Foteini Balomenou, Niki Dermitzaki, Chrisoula Kosmeri, Spyridon Giannakopoulos, Vasileios Giapros

**Affiliations:** Neonatal Intensive Care Unit, University of Ioannina, Faculty of Medicine, Stavrou Niarchou Avenue, 45500 Ioannina, Greece

**Keywords:** Interpretation, p Value, Statistical significance, Trend

## Abstract

Purpose This study is to examine whether the term “trend toward statistical significance” is used to describe statistically nonsignificant results in biomedical literature. We examined articles published in five high-impact pediatric journals, including *The Lancet Child & Adolescent Health*, *The Journal of Pediatrics*, *Early Human Development*, *Frontiers in Pediatrics*, and *BMC Pediatrics* to identify manuscripts where a “trend” was used to describe a statistically nonsignificant result, from January 2020 to December 2021, and, furthermore, for *The Journal of Pediatrics*, *Early Human Development*, and *BMC Pediatrics* from January 2010 to December 2011. We detected that a “trend toward significance” was used to describe a statistically nonsignificant result at least once in 146 articles (2.7%) during the period between 2020 and 2021 and in 97 articles (4.0%) during the period between 2010 and 2011. We found no significant difference in the proportion of published articles with inappropriate use of “trend” across journals belonging to the first quartile of impact compared to the second quartile or across journals publishing under the subscription model or open access policy compared to journals publishing solely under the open access policy, in any period. The overall proportion of the inappropriate use of “trend” declined significantly between 2010 and 2011 to 2020 and 2021 (*p* = 0.002, RR 0.66 95% CI 0.51–0.86).

*
Conclusion*: “Trend” statements were sporadically used to describe statistically nonsignificant results across pediatric literature. The inappropriate use of “trend” to describe almost significant differences could be misleading, and “trend” should be reserved only when a specific statistical test for trend has been performed, or in relation to appropriate scientific definitions.

**Table:** 

**What is Known:**
•*Previously, researchers have reported inappropriate use of “trend” in articles across anaesthesia or major oncology journals.*
•*In many cases, hypothesized results that are close but not lower than the statistical significance threshold are emphasized as “almost” significant.*
**What is New:**
•*“Trend” statements were sporadically used to describe statistically nonsignificant results across pediatric literature.*
•*Inappropriate use of “trend” was similar in journals with a subscription model compared to those having an open access policy and decreased within a 10-year period.*

## Introduction

In biomedical literature, reporting results that fail to reach statistical significance might be challenging [1]. When the *p* value approaches but does not reach the level of significance, authors commonly adopt the term “trend toward statistical significance” to refer to statistically nonsignificant results [1–4]; however, this inappropriate use of “trend” is problematic. In statistics, there is no definition of a “trend toward statistical significance” and, therefore, describing “almost significant” results as a “trend” might be misleading. In fact, when it is required to estimate the probability that observed changes in an apparent trend represent true rather than chance differences, formal statistical tests should be used including the chi-square test for linear trend, the Cochran-Armitage test, or the Mann–Kendall trend test [5–7]. Besides, “trend” can be used in the biomedical literature to describe apparent changes as per dictionary definitions.

Our aim was to examine whether the term “trend” was used inappropriately to describe statistically nonsignificant results, in articles published in high-impact pediatric journals.

## Materials and methods 

The selection of the journals was made selecting representative journals of the first and the second quartile of impact (Q1 and Q2 in the Scimago Journal & Country Rank database [8]). The two previously published articles examining the inappropriate use of “trends” in anesthesia [3], and oncology journals [1] have used a similar methodology. After the journal selection, all articles published during 2020–2021 and 2010–2011 were examined for inappropriate use of “trend.” The rationale of choosing those specific periods was that 10 years apart would be a sufficient period to examine if the establishment of the best statistical practice has been applied. We examined for inappropriate use of “trend” throughout the entire paper, including those articles that used the term “trend” to describe any statistically nonsignificant results.

We examined articles published in the journals *The Lancet Child & Adolescent Health*, *The Journal of Pediatrics*, *Early Human Development*, *Frontiers in Pediatrics*, and *BMC Pediatrics* to identify manuscripts where a “trend” was used to describe a statistically nonsignificant result, from January 2020 to December 2021, and for *The Journal of Pediatrics*, *Early Human Development*, and *BMC Pediatrics* from January 2010 to December 2011. We compared the results between the two periods, between journals belonging to the first (*The Lancet Child & Adolescent Health*, *The Journal of Pediatrics*, *Frontiers in Pediatrics*) and the second quartile of impact (*Early Human Development*, *BMC Pediatrics*) and between journals publishing under the subscription model (*The Lancet Child & Adolescent Health*, *The Journal of Pediatrics*, *Early Human Development*) and solely under the open access policy (*Frontiers in Pediatrics*, *BMC Pediatrics*).

### Statistical analysis

The proportion of the research articles with inappropriate use of “trend” across journals was compared by estimating percentages and 95% CIs. All tests were two-sided, and a *p* value less than 0.05 was considered statistically significant (alpha 0.05). The data were analyzed using SPSS Statistics (IBM SPSS Statistics for Windows, Version 24.0. Armonk, NY, USA). This project was exempt from approval by the Institutional Review Board because it was not human participants’ research.

## Results

We reviewed in total 5393 articles published during 2020–2021 and 2412 articles published during 2010–2011. A “trend toward significance” was used to describe a statistically nonsignificant result at least once in 146 articles (2.7%) during 2020–2021 and in 97 articles (4.0%) during 2010–2011. Although there were no significant differences in the proportion of the articles where a “trend toward significance” was used inappropriately across the different journals, the overall proportion of the articles that used “trend” inappropriately declined significantly between 2010–2011 and 2020–2021 (*p* = 0.002, RR 0.66 95% CI 0.51–0.86) (Table 1).

**Table 1:** Inappropriate use of trends across journals during the two periods

	**2020–2021**	**2010–2011**	***p* Value**	**95% CI**
*The Lancet Child & Adolescent Health*	0.2% (1/438)	-	-	-
*The Journal of Pediatrics*	2.8% (50/1798)	4% (58/1432)	0.061	0.68 (0.46–1.08)
*Early Human Development*	5.1% (21/412)	3.7% (28/750)	0.130	1.56 (0.87–2.79)
*Frontiers in Pediatrics*	2.4% (49/2004)	-	-	-
*BMC Pediatrics*	3.3% (25/743)	4.8% (11/229)	0.554	0.72 (0.55–1.94)
Total	2.7% (146/5393)	4% (97/2412)	0.002	0.66 (0.51–0.86)

*CI* confidence intervals

There was no significant difference in the proportion of articles with inappropriate use of “trend” across journals belonging to the first compared to the second quartile of impact during 2020–2021 (2.4% vs 4.0%, *p* = 0.083, RR 0.58 95% CI 0.40–1.08, respectively) or during 2010–2011 (4.0% vs 4.0%, *p* = 0.938, RR 1.01 95% CI 0.67–1.53, respectively). Finally, we recorded no significant difference across journals publishing under the subscription model compared to journals publishing under the open access policy during 2020–2021 (2.7% vs 2.7%, *p* = 0.954, RR 1.01 95% CI 0.72–1.40, respectively) or during 2010–2011 (3.9% vs 4.8%, *p* = 0.537, RR 0.81 95% CI 0.53–1.55, respectively).

## Discussion

In the current study, we found that “trend” statements were used to describe statistically nonsignificant results across pediatric literature. In most of the cases, the term “trend” was used inappropriately to describe a *p* value that was close but not smaller than the level of 0.05 that typically defines statistical significance.

Applying the term “trend” to report an almost significant result is a common pitfall in biomedical literature. Previously, researchers have reported inappropriate use of “trend” in 3.4% of articles across anesthesia journals [3], and 8.7% of articles in major oncology journals [1]. Interestingly, the inappropriate use of “trend” was consistent across journals of different impacts, across different periods, and, as shown in our study, across journals of different publication policies. Of note, our findings suggested that the overall proportion of the inappropriate use of “trend” across pediatric journals declined significantly between 2010 and 2011 to 2020 and 2021, which might indicate that, during the last decade, statistical interpretation has become more robust. Several journals currently include articles and tutorials helping authors to submit manuscripts with more precise statistics, while we believe that many young researchers are better qualified in medical statistics through several courses and seminars [9]. Besides, based on our findings, the inappropriate use of “trend” across pediatric journals was rarer compared to other fields of medicine, especially in oncology, whereas there was similar to the field of anesthesia.

According to the fundamentals of statistics, a *p* value is either significant or non-significant [10]. In statistics, a *p* value, or probability value, describes how likely the data would have occurred by random chance, i.e., the *p* value represents the evidence against a null hypothesis. The outcome of an inferential test is either rejection of the null hypothesis or failure to reject the null hypothesis [11]. There is no other option, and therefore a statement of “trend of significance” would be misleading.

In many cases, hypothesized results that are close but not lower than the statistical significance threshold are emphasized as “almost” significant. The use of “trend” to describe statistically nonsignificant results commonly arises from the authors’ intention to report their findings suggesting that the nonsignificant differences they observed might have been significant if the sample size increased. However, as suggested by Wood et al., a *p* value is by no means assured to become smaller even with the addition of quite a substantial proportion of extra data, a finding that could not support any claim of a “trend” towards statistical significance [4]. Although it has been demonstrated that underpowered studies could miss true findings, on the other hand, a failure to demonstrate statistical significance is not always due to sample size [4]. Moreover, statistical significance should be interpreted separately from clinical significance. Even statistically significant results might be meaningless in clinical practice, especially if the analysis does not include estimates and confidence intervals for effect sizes [10]. Besides, to address such issues, the American Statistical Association has released recommendations suggesting that scientific conclusions should not be based solely on a *p* value threshold and that a *p* value does not measure the importance of a result and does not provide a good measure of evidence regarding a hypothesis [11].

Although we limited our research to five pediatric journals, we included journals of a high impact, published in different regions, with a broad readership. We, therefore, have no reason to believe that our findings do not reflect a general issue in the pediatric literature.

In summary, we found that “trend” statements were sporadically used to describe statistically nonsignificant results across pediatric literature. However, the inappropriate use of “trend” to describe almost significant differences could be misleading as it could be falsely interpreted as suggesting a real trend. Therefore, “trend” should not be used to describe non-significant statistical differences, unless a specific statistical test for trend has been performed, or in relation to appropriate scientific definitions.

## Data Availability

Not available.
